# 3D-printed microfibers encapsulating stem cells in scaffold with tri-culture and two-stage metformin release for bone/vasculature/nerve regeneration in rats

**DOI:** 10.1016/j.bioactmat.2025.05.011

**Published:** 2025-05-21

**Authors:** Minjia Zhu, Xinyi Li, Le Xiao, Kan Yu, Jingyi Li, Zixiang Dai, Qinrou Zhang, Jialiang Dai, Zihan Jia, Yuxing Bai, Ke Zhang

**Affiliations:** a Department of Orthodontics, Beijing Stomatological Hospital, School of Stomatology, Capital Medical University, Beijing, 100070, PR China; b Department of Orthodontics, Shanghai Stomatological Hospital and School of Stomatology, Fudan University, Shanghai, 200001, PR China; c Department of Dentistry, Beijing Friendship Hospital, Capital Medical University, Beijing, 100050, PR China

**Keywords:** Bone tissue engineering, Innervation, Osteogenesis, Tri-culture, Metformin

## Abstract

**Introduction:**

Regeneration of critical-sized bone defects remains a major clinical challenge. Solely promoting osteogenesis is inadequate, because vasculature and neural innervation are critical for establishing the bone regenerative microenvironment.

**Objective:**

For the first time, the present study developed 3D bio-printed hydrogel microfibers (aMF) encapsulating human periodontal ligament stem cells (hPDLSCs) in a tri-culture system in calcium phosphate cement (CPC) scaffold with a two-stage metformin release for regeneration of nerve, vasculature, and bone.

**Materials and methods:**

This tri-culture system consisted of hPDLSCs, human umbilical vein endothelial cells (hUVECs), and pericytes (PCs). Moreover, we employed 3D-bioprinted aMF in CPC scaffold with a controlled two-stage release system for metformin release to promote bone, vasculature, and nerve regeneration.

**Results:**

Our innovative construct increased the regenerated amounts of bone, vasculature and nerve significantly by 2.5-fold, 3-fold, and 3.5-fold, respectively, than control group, in cranial defects in rats.

**Conclusion:**

This novel hPDLSCs tri-culture system in aMF-CPC scaffold with two-stage metformin release is highly promising for the regeneration of all three tissues of bone, vasculature, and nerves in a wide range of craniofacial and orthopedic applications.

## Highlights

• The tri-culture system in aMF-CPC scaffold with two-stage metformin release promotes bone, vasculature and nerve regeneration.

## Graphical abstract

**Figure:**
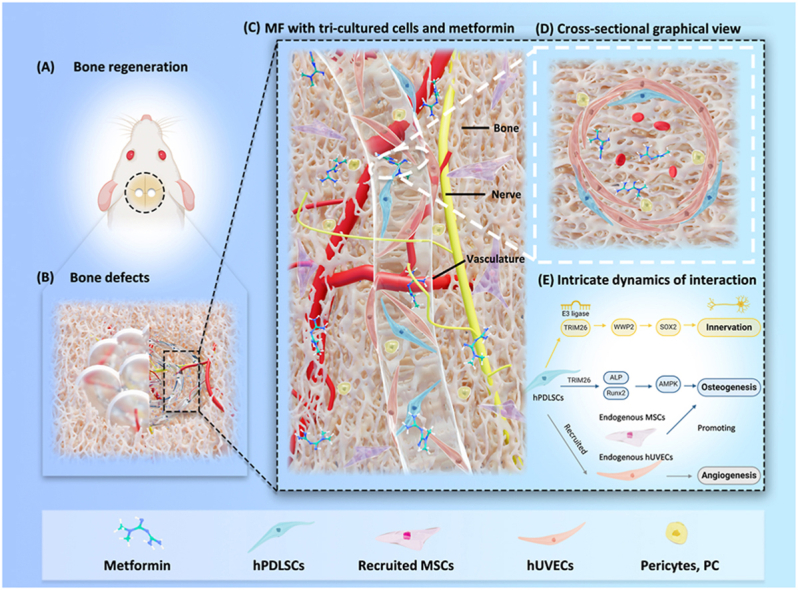

## 1 Introduction

Critical-sized bone defects, often resulting from trauma, tumour, inflammation, or surgical interventions, compromise not only the structural integrity of the skeleton but also impede normal physiological functions and the quality of life [[1], [2], [3]]. The complexity of bone tissue, characterized by its mechanical properties and intricate architecture with inner vasculature and nerves, renders spontaneous regeneration challenging, often leading to prolonged disability and impaired mobility [4,5]. Repairing bone defects is particularly arduous due to the necessity for concomitant regeneration of both vasculature and nerve, which play critical roles in supporting osteogenesis. Vascularization is essential for delivering nutrients, oxygen, and signalling molecules necessary for bone healing, while neural regeneration is pivotal for restoring functional connectivity and mediating pain management [6,7]. Despite the recognized importance of these interrelated processes, there remains a notable lack of comprehensive research addressing the integrated regeneration of bone, vasculature, and nerves. To address these challenges, developing advanced tissue engineering scaffolds designed to facilitate the integrated repair of bone, vasculature, and nerves presents a promising avenue. Such scaffolds could incorporate seed cells and growth factors for bone regeneration and ultimately improve the clinical outcomes for patients suffering from critical-sized bone defects [8,9]. However, most scaffolds fail to provide a sustained, long-term, controlled release pattern.

Three-dimensional (3D) bio-printing technology can provide a personalized approach to fabricating controlled structures in regenerative medicine [10]. In our previous studies, we constructed a stacked alginate hydrogel scaffold characterized by different degrees of oxidative substitution and capable of degrading at a specified time interval [[11], [12], [13]]. 3D bio-printed alginate hydrogel microfibers (aMF) exhibit advantageous degradation properties compared to the oxidized stacked alginate hydrogel scaffolds; moreover, they possess an even better distribution ability of the encapsulated cells and bioactive factors. Therefore, our 3D bio-printed can be an ideal carrier in developing the microenvironment of the long-term osteogenesis process. Until now, no reports have been published on the construction of 3D bio-printed tissue engineering scaffolds focusing on tri-dimensional tissue repair: bone vasculature and nerve.

Introducing bioactive factors is essential to stimulate the physiological responses of the tissue repair process. Previous studies have found that bone morphogenetic protein 2 (BMP2), an FDA-approved bone regenerative growth factor, could enhance the repair of skull bone defects [14]. Others have added transforming growth factor-β (TGF-β) into the scaffold to promote bone regeneration [15]. However, these classical growth factors demonstrate limited capabilities beyond osteogenesis and do not satisfy safety standards when administered directly into the microenvironment. In contrast, metformin, a classical antidiabetic agent that is recognized as the standard treatment for type II diabetes mellitus, is proven to have new potential in the field of bone regeneration [16,17]. In addition to its osteogenesis effects, metformin has been proven to be able to protect the cardiovascular system and promote angiogenesis and pre-vascularization [18]. Therefore, we introduced metformin into the aMF-CPC controlled release system. The two-stage release system is designed to establish a controlled and even release profile, thereby fostering an optimal bone regenerating microenvironment. This system is capable of delivering essential bioactive factors during stage one, prior to the release of cells, and subsequently co-releasing these factors alongside the cells at stage two. In summary, the two-stage release of metformin significantly enhances the three-dimensional bone regeneration process.

Although scaffolds containing metformin have been reported to effectively regulate the bone microenvironment and promote the regeneration of bone defects [19], there are still limitations due to their inability to encourage both nerve and vascular ingrowth. Co-culture systems are designed to enhance the interactions among various cell types [20]. In contrast to mono-culture, coculture or triculture cells may exhibit exceptional responses. The tri-culture system, in particular, could accurately mimic the bone microenvironment, promoting physiological processes related to vascular and nerve induction. The tri-culture system consists of human periodontal ligament stem cells (hPDLSCs), human umbilical vein endothelial cells (hUVECs), and pericytes (PCs). HPDLSCs are easily harvested mesenchymal stem cells (MSCs) and could differentiate into multiple cell lines [21]. HUVECs and PCs play a critical role in the maturation process of the pre-vascularized vessels [22,23]. The integration of these tri-culture cells, characterized by complex interaction dynamics [24], may potentially enhance the simulation of bone repair. Therefore, within the microenvironment established by the metformin released from the two-stage controlled aMF-CPC scaffold, the tri-culture cells may possess the capacity to enhance the maturation of endogenous capillaries and the corresponding neural structures.

**Fig. 1:**
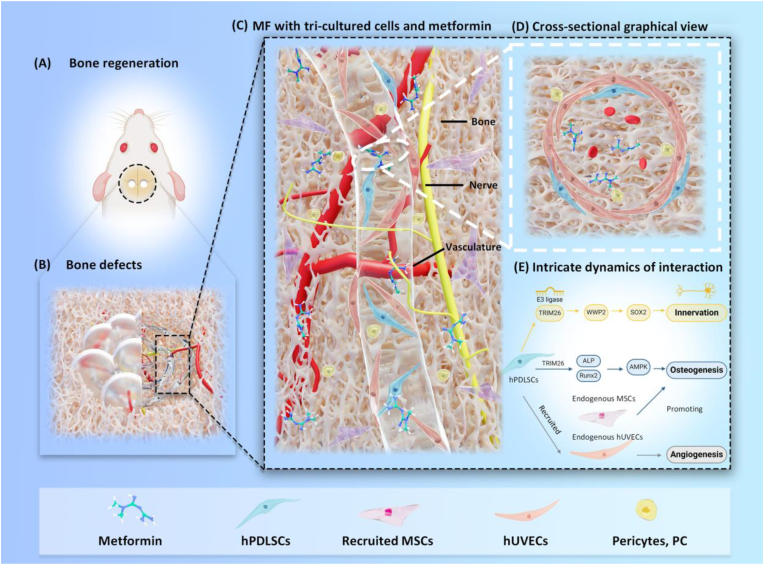
Schematic of the study design. (A) 3D bio-printed aMF-CPC scaffold has demonstrated the capability to repair critical-sized bone defects. (B) 3D bio-printed aMF-CPC scaffold can promote bone, vascularization, and nerve tissue repair. (C) The 3D bio-printed hPDLSCs-based tri-culture system. This tri-culture system is based on hPDLSCs and consists of human umbilical vein endothelial cells (hUVECs) and pericytes (PCs). (D) The cross-sectional graphical view of the ingrowth vessel containing tri-culture cells. (E) The intricate dynamics of interaction transpired within the tri-culture systems.

A literature search revealed no report on the development of a two-stage metformin release system for sustained enhancement of regeneration of bone, vasculature and nerve tissues. This study presents the first report of a 3D bio-printed aMF encapsulating hPDLSCs-based tri-culture system in CPC scaffold with two-stage metformin release. We have innovatively constructed this tri-culture cellular system encapsulated in the aMF-CPC scaffold to promote bone tissue regeneration (Fig. 1A–and C).

This study aimed to (1) develop for the first time an hPDLSCs-based tri-culture system to promote bone, vasculature and nerve regeneration simultaneously; (2) construct an innovative 3D bio-printed aMF encapsulating hPDLSCs-based tri-culture system in CPC scaffold with two-stage metformin release; (3) investigate the promotion of bone, vascularization, and nerve tissue repair over temporal and spatial dimensions in a rat cranial bone defect model and its primary mechanism for the first time. The following hypotheses were evaluated: (1) 3D bio-printed aMF encapsulating hPDLSCs-based tri-culture system would demonstrate superior ability for promoting capillary maturation compared to mono- or co-culture systems, with metformin being released from the scaffold at controlled two-stages; (2) 3D bio-printed aMF encapsulating hPDLSCs-based tri-culture system in CPC scaffold with two-stage metformin release would have the potential to enhance bone formation as well as vascular and nerve ingrowth, both *in vitro* and *in vivo*; (3) the potential ingrowth of capillaries and nerves would be derived from and regulated by protein TRIM26.

## 2 Methods

### 2.1 Cell culture

HPDLSCs were harvested through the middle third tissues of the premolars extracted from healthy patients aged 18–22 [25]. The Ethics Committee of Beijing Stomatological Hospital, affiliated with Capital Medical University, China, approved this experiment. HUVECs and PCs were purchased and used in passages 3–5 for the following experiments. The tri-culture medium consisted of the supplementation of each cell component. The proportions of each endothelial cell medium (ECM) and pericyte cell medium (PCM) were consistent with the cellular ratios established within the tri-culture system. Metformin was added to the medium at 0 mg/mL, 100 mg/mL, and 200 mg/mL. After cultivating primary hPDLSCs, flow cytometry was conducted to characterize these cells. Additionally, immunofluorescence staining was performed to assess the expression of PECAM1 and Desmin in hUVECs and PCs.

### 2.2 Fabrication of 3D bio-printed aMF in CPC scaffold

The bioink suspension (as detailed in Table S1) comprised a tri-culture of cells totalling 1 × 10^6^, with a composition ratio of hPDLSCs, hUVECs, and PCs set as 4:12:1. Additionally, the bioink also included 200 mg/mL metformin and alginate phosphate. Subsequently, the bioink was introduced into the needle tube of the printer (Allevi, USA). The detailed FRESH process was illustrated in Movie S1. As previously reported [26], the parameters of needle diameter, printing speed, and extrusion pressure were maintained at constant values of 27 G, 10 mm/s, and between 1.5 and 1.8 kPa, respectively, during the printing procedure. The CPC scaffold was synthesized using a combination of tetra calcium phosphate, dicalcium phosphate anhydrous, and a chitosan solution, incorporating 200 mg/mL of metformin. Then, the 3D bio-printed aMF was assembled within the CPC scaffold to establish the two-stage release system.

### 2.3 Mechanical properties of the scaffolds

The CPC scaffold underwent evaluation using FTIR and atomic force microscope (AFM). The 3D bio-printed aMF-CPC scaffold was also analyzed using SEM following a 21-day incubation cycle. Furthermore, the mechanical characteristics of the CPC-aMF scaffolds were assessed with a universal testing machine, focusing on flexural strength, elastic modulus, and cyclic loading tests. To evaluate the long-term fatigue performance of the CPC and CPC-aMF scaffolds, compression was applied in the direction of the scaffolds' thickness. The displacement rate was set at 0.5 mm/min, with compressive strain reaching up to 30 % of the initial thickness before being released. The load and unload curves for the 1st-10th cycles were recorded.

The amount of metformin released from the two-stage scaffolds was analyzed using high-performance liquid chromatography (Thermo Fisher Scientific, USA), as reported [27]. The concentration of metformin was then quantified using a standard curve along with the measured data. Additionally, pH changes and *in vitro* cytotoxicity during the degradation of the scaffolds were assessed.

To assess the mechanical properties and illustrate the advantages of two-stage release scaffolds, the following groups were subjected to testing:

① Blank control (CPC scaffold only)
② Negative control (CPC-aMF with 0 MET control)
③ CPC-aMF with 200 mg/mL MET in CPC
④ CPC-aMF with 200 mg/mL MET in aMF
⑤ CPC-aMF with 200 mg/mL MET in both
⑥ Cancellous bone (bone control)

### 2.4 In vitro experiments

Tri-culture cells were induced to undergo osteogenic differentiation for 1, 7, 14, and 21 days. After the assessment of ALP and ARS staining along with semi-quantification, qRT-PCR was employed to evaluate the transcription levels of osteogenic genes (primers are as detailed in Table S2). The CCK-8 assay for cell proliferation was performed on mono-, co-, and tri-cultured cells.

### 2.5 Animal experiment

The Animal Ethical Committee of Beijing Stomatological Hospital, affiliated with Capital Medical University, China, approved the animal experiment. The Sprague-Dawley (SD) rats were randomly assigned to five groups (both sides of the cranial bone were used):

① Blank control (CPC only with 0 tri-culture cells, 0 MET blank)
② Negative control (CPC-aMF with 0 tri-culture cells, 0 MET control)
③ CPC-aMF with 0 tri-culture cells, 200 mg/mL MET
④ CPC-aMF with tri-culture cells, 0 MET
⑤ CPC-aMF with tri-culture cells, 200 mg/mL MET in both

The *in vivo* study involving critical-sized cranial defect models used male SD rats aged 8 weeks. After applying anesthesia and implementing sterilization protocols, critical-sized defects measuring 5 mm in diameter were created bilaterally in the cranial bone using a circular drill. After the implantation of scaffolds, the incision in the skull was sutured shut, and antibiotics were then provided [28]. Cranial bone tissues were harvested after 1, 4, 8, and 12 weeks, followed by the assessment of Micro-CT imaging, HE staining, and Masson staining. Additionally, immunohistochemical and immunofluorescence staining techniques were employed to assess inflammatory factors (IL-1β and TNF-α), angiogenic markers (CD31 and α-SMA), and innervation markers (CGRP and β3-Tubulin), following established protocols.

### 2.6 RNA sequencing analysis

Total RNA was isolated from the samples using the TRIzon Reagent kit (Cowin Biotech, China) according to the manufacturer's instructions. The quality and concentration of the RNA were then evaluated. Based on Veen diagram, differentially expressed genes, a volcano plot, heatmap, KEGG, and GO enrichment analysis were conducted. PPI networks of TRIM26 were then analyzed using the STRING database, with adjustments made according to a stringent threshold established by the Q value.

### 2.7 Western blot

This procedure was employed to evaluate the expression of TRIM26, and its associated signalling pathways in regulating nerve and bone tissue repair. The proteins TRIM26, Runx2, and MAPK were analyzed across these experimental groups: Control, Agonist, Inhibitor, Overexpress, and Knockdown (n = 3). The groups subjected to protein expression analysis included:

① MET (200 mg/mL MET)
② Agonist (200 mg/mL MET with 100 μM TRIM26-cAMP agonist Forskolin)
③ Inhibitor (200 mg/mL MET with 20 μM TRIM26-MDM2 inhibitor Nutlin-3)
④ Overexpress (200 mg/mL MET with shRNA for TRIM26 gene overexpression)
⑤ Knockdown (200 mg/mL MET with shRNA for TRIM26 gene knockdown)

Human TRIM26 (NM_003449.5) was obtained from the cDNA library using the primers (as detailed in Table S2). The vector plasmid GV657, and the TRIM26 gene sequence underwent digestion with BamHI and KpnI restriction enzymes, followed by complete cloning via the in-fusion recombination method. Furthermore, the plasmid vector designed to express shRNA targeting the TRIM26 gene sequence (as detailed in Table S2) was synthesized and subsequently cloned into the GV493 vector at AgeI and *Eco*RI restriction sites. The shRNA-TRIM26 plasmid was then extracted using the Endofree plasmid Mega kit from Escherichia coli. The TRIM26 overexpression and knockdown plasmid were then applied to tri-cultured hPDLSCs with 200 mg/mL metformin.

Proteins were extracted from the cellular samples, and the primary antibodies were anti-TRIM26, anti-Runx2 and anti-MAPK (Cell Signaling Technology). The resulting bands from the Western blotting were subsequently analyzed using ImageJ software. Protein levels were standardized against GAPDH as a reference.

### 2.8 Statistical analysis

The data obtained were statistically analyzed using SPSS software (version 21.0) and were presented as mean ± standard deviation (SD). All experiments were performed with four replicates *in vitro* and four *in vivo*. T-tests and one-way ANOVA multiple comparisons were analyzed among different groups. The significance level was set at ∗*P* < 0.05, ∗∗*P* < 0.01, and ∗∗∗*P* < 0.001.

## 3 Results and discussion

### 3.1 The design and fabrication of the 3D bio-printed aMF

**Fig. 2:**
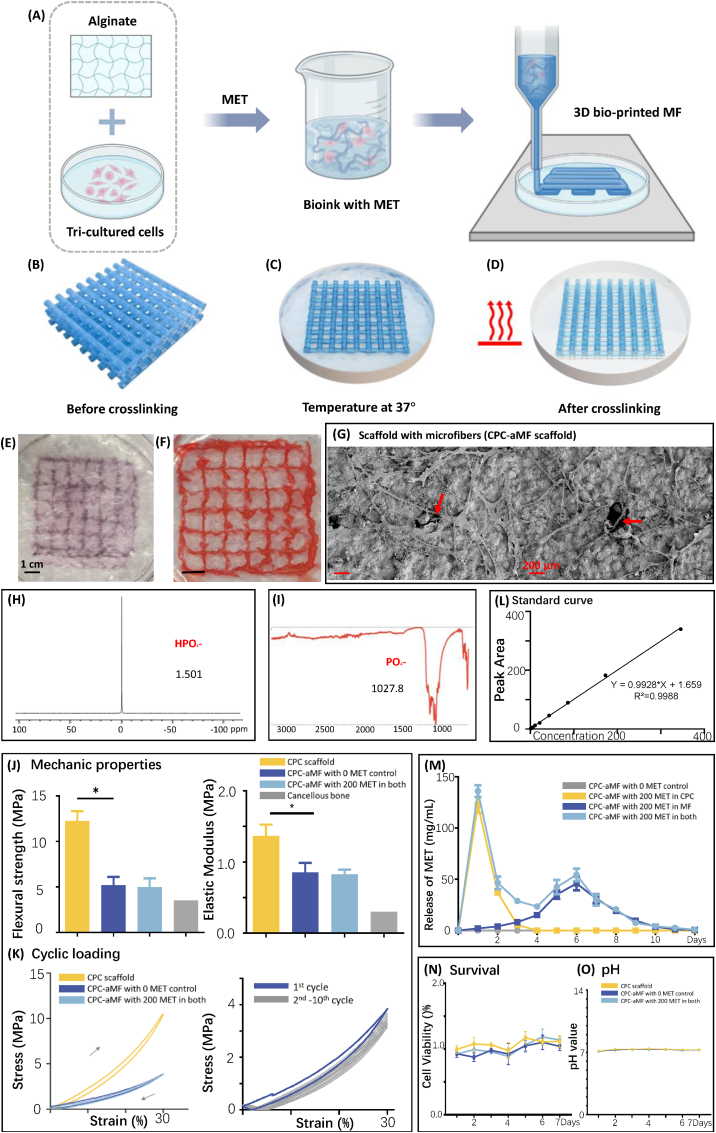
3D bio-printed aMF encapsulating hPDLSCs-based tri-culture system in CPC scaffold for extensive bone repair. (A) Illustrates the design and composition of the bioink. (B) Computer-aided 3D design model. (C–D) Schematic representations of a 3D bio-printed aMF carrier system using the FRESH technique. (E–F) Gross appearance of the 3D bio-printed aMF. Scale bar = 1 cm (G) SEM images illustrate the presence of macropores that enable the extension of tri-culture cells (see arrow). Scale bar = 200 μm. (H) FTIR showing PO_4_^3^^−^ in the CPC scaffold. (I) Atomic force microscope showing HPO_4_^2^^−^ in the CPC scaffold. (J) The scaffolds' flexural strength and elastic modulus assessments. (K) The load-unload curves of the scaffolds. (L) The standard curve of metformin concentration. (M) Metformin release curve of the aMF-CPC scaffold. (N) Cell cytotoxicity test throughout the degradation process of the scaffolds. (O) pH changes throughout the degradation process of the scaffolds.

Inspired by the exceptional matrix viscoelastic properties of 3D-bioprinted hydrogel [29], an alginate hydrogel carrier system was developed and fabricated through the FRESH (Freeform Reversible Embedding of Suspended Hydrogels) technique [26] **(**movie S1**)**. Fig. 2A illustrates the design and composition of the bioink (Table S1). The alginate hydrogel was designed to have an oxidation level of 7.5 %, which was optimal for facilitating the controlled degradation rate. In the tri-culture system, the ratio of hPDLSCs, hUVECs, and PCs was designed to be 4:12:1 (Fig. S1), a proportion that is essential for promoting interactions pertinent to bone formation. Metformin, recognized for its excellent osteogenesis performance in bone tissue engineering, was added to the bioink at 200 mg/mL concentration. The diameter of the alginate microfibers was designed as 200 μm, an aligned diameter that not only supports the ingrowth of nerves and vessels but also enhances oxygen penetration necessary for capillary formation. Bioink was introduced into a projection-based 3D bio-printer, which enabled the construction of a biomimetic scaffold (Fig. 2B) by cross-linking alginate microfibers with calcium ions (Ca^2+^) in the suspension bath. After incubation at 37 °C (Fig. 2C–and E), the residual bath liquid was removed (Fig. 2D–and F).

The cellular biocompatibility of the aMF-CPC scaffold was characterized by scanning electron microscopy (SEM). SEM images revealed an even distribution of the tri-culture cells, which exhibited an elongated morphology. Additionally, macropores were formed upon degradation of the 3D bio-printed aMF (see arrow). These macropores (100–300 μm in diameter) enabled the extension of cells (Fig. 2G), thereby promoting the ingrowth of vessels and nerves.

**Fig. 3:**
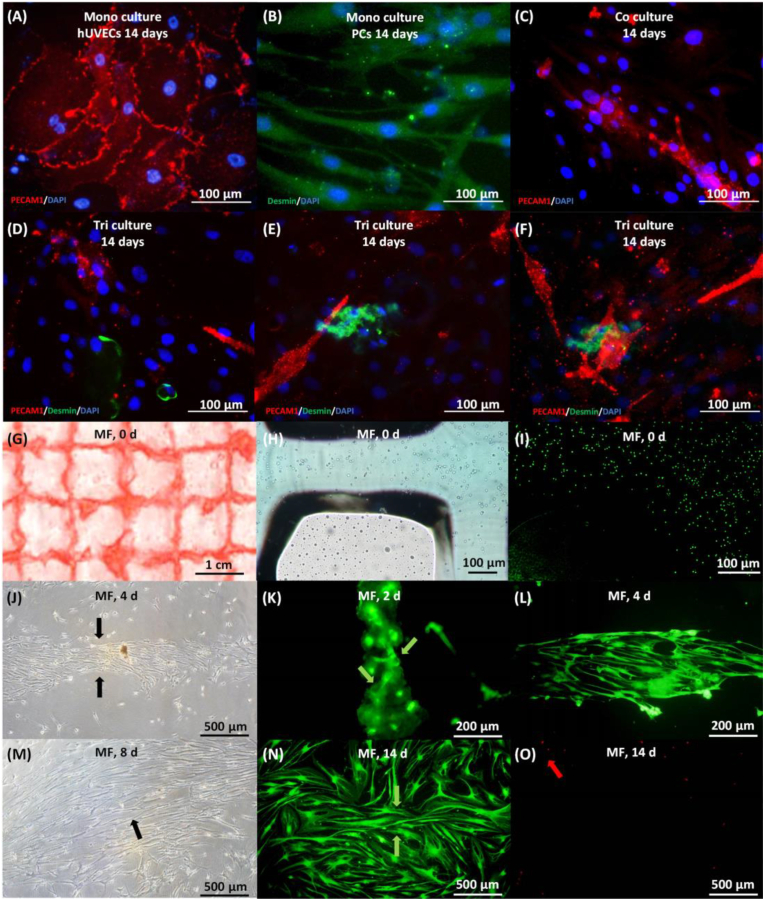
Characterization of 3D bio-printed aMF encapsulating hPDLSCs -based tri-culture system. (A–B) Immunofluorescence images of mono-cultured hUVECs and PCs. Scale bar = 100 μm. (C) Immunofluorescence images of co-cultured hPDLSCs and hUVECs. Scale bar = 100 μm. (D–F) Immunofluorescence images of tri-culture hPDLSCs, hUVECs, and PCs. Scale bar = 100 μm. (G) Gross appearance of the 3D bio-printed aMF encapsulating hPDLSCs -based tri-culture system. Scale bar = 1 cm (H, J, M) Microscopic images of the 3D bio-printed aMF at days 0, 4, and 8 illustrate the proliferation of hPDLSCs following the programmed degradation of the microfiber (see arrow). Scale bar = 100 μm and 500 μm. (I, K-L, N-O) Live/dead assay of the 3D bio-printed aMF at days 0, 2, 4, and 14, illustrating the proliferation of hPDLSCs (with green arrows indicating live cells and red arrows denoting the dead cells). Scale bar = 100 μm, 200 μm and 500 μm.(For interpretation of the references to colour in this figure legend, the reader is referred to the Web version of this article.)

The structure of aMF might also provide cellular protection during the 3D bio-printing procedure. The visual assessments of gross appearance, microscopic images and the results from the live/dead assay conducted via fluorescence microscopy suggested that the protective measures implemented by aMF are effective and that the 3D bio-printing process does not induce cellular damage (Fig. 3G–and I). Thus, a hPDLSCs-based tri-culture system encapsulated in aMF with controlled metformin release was successfully fabricated through 3D bio-printing technology.

### 3.2 The characterization of the aMF-CPC-controlled two-stage release system

CPC was introduced into the scaffold system to mimic the mechanical properties of native craniofacial bone and establish a controlled release system. Initially, CPC contributed to the scaffold's structural integrity [30]. CPC was synthesized by our prior research findings [25]. The constitution and structural characteristics of the CPC were verified through Fourier-transform infrared spectroscopy (FTIR) and atomic force microscope (Fig. 2H–and I).

The mechanical properties of the aMF-CPC scaffold were then evaluated. The excellent mechanical characteristics of CPC, which are advantageous for facilitating the biomechanical functions necessary for bone tissue regeneration, were not diminished by the incorporation of the 3D bio-printed aMF. Both the flexural strength and elastic modulus of the CPC-aMF scaffold were found to be significantly greater than those of cancellous bone (Fig. 2J). In order to further investigate and simulate the long-term application of the scaffold *in vivo*, cyclic loading experiments were performed. The stress-strain characteristics of the scaffolds were then assessed, and the load-unload behaviours were recorded. Notably, after the 10th cycle of loading, the CPC-aMF scaffold exhibited no significant deterioration in fatigue performance compared to the 2nd cycle (Fig. 2K).

Furthermore, CPC has the potential to function as an auxiliary vehicle for the delivery of growth factors. In our controlled two-stage release system, metformin was encapsulated in both aMF and CPC. During stage one, metformin was released from the CPC, promoting the recruitment of endogenous MSCs and related bifactors to establish a preliminary microenvironment conducive to bone formation. This initial stage occurred early, on days 1 and 2. The confocal microscopy images indicated that elongated and fusion-formed tri-culture cells were observed on days 2 and 4, promoted by the preliminary bone-forming microenvironment (Fig. 3K–and L). As illustrated by the standard curve of metformin concentration (Fig. 2L), and metformin release curve, in the CPC group (CPC-aMF with 200 MET in CPC), the concentration of metformin peaked on day 1 and continued its release until day 4 (Fig. 2M). In addition, to assess cellular viability throughout the degradation process of the scaffolds, measurements of pH changes and cytotoxicity were conducted. The observed cell survival rates, along with pH values ranging from 7.2 to 7.4, indicate that the degradation products of the CPC-aMF scaffold do not compromise the structural integrity of aMF or the cellular viability of the tri-culture system (Fig. 2N–and O).

In stage two, metformin and the tri-culture cells were designed to be released from the aMF, which was designed to undergo a predetermined degradation process. Stage two started on day 4, when the initial microenvironment was established to promote cell growth, differentiation, and secretion of the tri-culture cells and enhance their interactions. As the degradation of the aMF progresses, the tri-culture cells exhibited a gradual increase in proliferation at days 4 and 8, promoted by the microenvironment established during early stage one and later at stage two (Fig. 3J–and M). Furthermore, the metformin release curve indicated that the aMF group (CPC-aMF with 200 MET in aMF) reached its peak release at day 6, after which all groups completed their release by day 12 (Fig. 2M). Eventually, on day 14, all tri-culture cells exhibited excellent proliferation and demonstrated minimal cytotoxicity when observed under a confocal microscope (Fig. 3N–and O). These suggested the potential role of tri-culture cells as centers for ossification and capillary formation, which might promote subsequent processes of osteogenesis and angiogenesis.

In summary, our controlled two-stage release system of the aMF-CPC scaffold can release metformin and seed cells at a sustained rate. In contrast to traditional stacked hydrogel scaffolds, the aMF-CPC-controlled two-stage system offers a solution throughout the osteogenesis cycle.

These groups were tested: Blank control (CPC scaffold only), Negative control (CPC-aMF with 0 MET control), CPC-aMF scaffold with 200 mg/mL MET in both (controlled two-stage release of MET), cancellous bone (bone control), CPC-aMF scaffold with 200 MET in CPC, and CPC-aMF scaffold with 200 MET in MF.

All values were presented as the mean ± SD, ∗*P* < 0.05, ∗∗*P* < 0.01, and ∗∗∗*P* < 0.001 analyzed by one-way ANOVA (n = 4).

### 3.3 The physiochemical properties of the tri-culture system

**Fig. 4:**
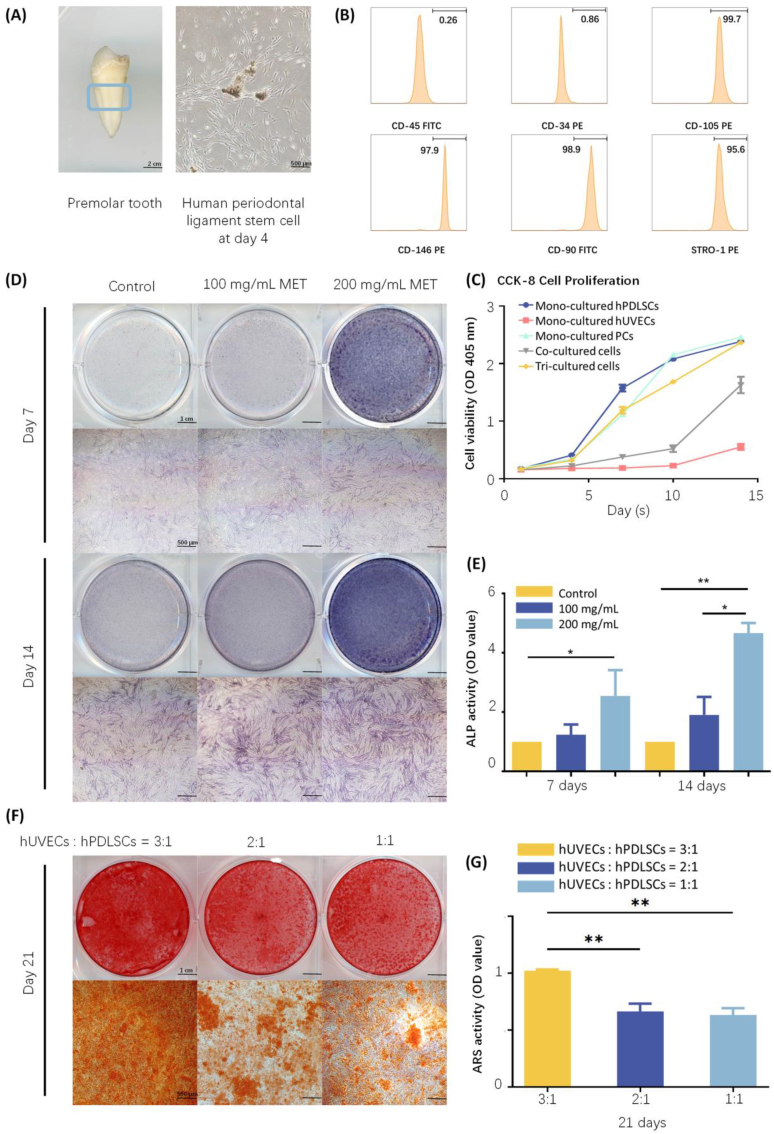
Osteogenic differentiation activity of tri-culture cells with metformin *in vitro*. (A) PLCs were easily obtained and cultured from human dental tissues. (Scale bar = 2 cm and 500 μm) (B) Flow cytometry of stem cell hPDLSCs. (C) CCK-8 cell proliferation rate of tri-culture cells for 1,4,7 and 14 days (n = 4). (D) Gross view and microscopic images of ALP staining of tri-culture cells with 0 mg/mL, 100 mg/mL, and 200 mg/mL metformin at days 7 and 14. (Scale bar = 1 cm and 500 μm) (E) Effects of different concentrations of metformin on ALP activity in tri-culture cells at days 7 and 14 (n = 4). (F) Gross view and microscopic images of ARS staining for various ratios of tri-culture cells at day 21. (Scale bar = 1 cm and 500 μm) (G) ARS activity (semi-quantification of cell synthesized minerals) of various ratios of tri-culture cells (n = 4). All values were presented as the mean ± SD, ∗*P* < 0.05, ∗∗*P* < 0.01, and ∗∗∗*P* < 0.001 analyzed by one-way ANOVA (n = 4).

The tri-culture system systematically integrated three cell types: hPDLSCs, hUVECs, and PCs. HPDLSCs, classified as traditional MSCs, have the potential to serve as a valuable resource for bone regenerative therapies [31]. Compared to other MSCs, such as bone marrow stromal cells, hPDLSCs exhibit a similar capacity for differentiation into various lineages, including osteoblasts, hematopoiesis-supportive stroma, and chondrocytes. In addition, hPDLSCs can be easily obtained and cultured from human dental tissues (Fig. 4A–and B). hUVECs, which represent an endothelial cell line [32], have the potential to secrete vascular endothelial growth factor (VEGF), thus promoting the process of angiogenesis. When mono-cultured, hUVECs exhibited a high platelet endothelial cell adhesion molecule 1 (PECAM1) expression, as demonstrated by immunofluorescence staining (Fig. 3A).

Nevertheless, capillary-like structures were not detected in the mono-culture groups. On the contrary, the co-culture of hUVECs and hPDLSCs groups exhibited a pronounced formation of capillary-like structures, as observed through confocal microscopy (Fig. 3C). These phenomena may be attributed to the interactions occurring within the co-cultured system. Nanovesicles, nanoparticles, and bioactive factors derived from hPDLSCs were released into the co-cultured microenvironment [33]. These vesicles contained multifunctional therapeutic cytokines that had the potential to augment both osteogenesis and angiogenesis. This enhancement might be synergistically promoted by factors derived from hUVECs, creating a positive feedback mechanism.

Pericytes play a crucial role in the regulation of capillary function and the maturation of blood vessels [34]. When mono-cultured, PCs demonstrated a high expression of Desmin protein, as evidenced by immunofluorescence staining (Fig. 3B). Upon introducing PCs into the co-cultured system, Desmin was detected in conjunction with the capillary-like structures, suggesting a tendency for migration and a protective function (Fig. 3D–and E). Compared to the co-cultured system without PCs, a more significant proportion of PECAM1 was observed (Fig. 3F). These findings indicated that PCs might play an essential role in promoting angiogenesis. Furthermore, the optimal cell ratio within the tri-culture system will be further analyzed in the following sections.

In conclusion, the interactions within tri-culture systems are complex and not yet well-defined. The physiochemical mechanisms underlying tri-culture cells are still inadequately understood, partly due to the limited number of *in vivo* and *in vitro* studies performed [35]. The tri-culture system will be introduced into our aMF-CPC scaffold system in the following sections.

### 3.4 Metformin and tri-culture system incorporation induced osteogenic activity *in vitro*

Based on our previous experimental findings, metformin demonstrated a beneficial capacity for bone formation on hPDLSCs at a concentration of 200 mg/mL. This present study aims to examine the influence of metformin on the osteogenic and angiogenic characteristics of all tri-culture cells.

The cell proliferation rate was assessed using a CCK-8 assay, and the results were presented as the optical density (OD) at 405 nm values. At day 14, the proliferation rate of hPDLSCs was three times greater than that of the hUVECs. Moreover, the proliferation rate observed in the co-culture system was intermediate between that of the individual cell types. The proliferation rate of the PCs did not exhibit a significant difference compared to that of the hPDLSCs. The proliferation rate of the tri-culture system would likely fall between the rates observed for the three individual cell types. However, the findings indicated that this rate was not significantly lower than that of the hPDLSCs (Fig. 4C). These findings might be attributed to a specific proportion of all three cell types.

At a metformin concentration of 200 mg/mL, various ratios of the tri-culture system were tested. In a co-cultured system involving hPDLSCs and hUVECs, the 1:3 ratio groups exhibited a higher mineral matrix formation capacity compared to the 1:2 and 1:1 ratio groups, as assessed by the alizarin red staining (ARS) method (Fig. 4F–and G). Subsequently, in a co-culture system containing hPDLSCs and PCs, the same experiments were conducted, revealing that the ideal ratio of hPDLSCs to PCs was 4:1. In conclusion, the perfect ratio for the tri-culture system, consisting of hPDLSCs, hUVECs, and PCs was determined to be 4:12:1.

Subsequently, the osteogenic potential of tri-culture cells, stimulated by different concentrations of metformin, was assessed by measuring alkaline phosphatase (ALP) activity at days 7 and 14. Fig. 4D–and E illustrated that the 200 mg/mL group demonstrated a 2-fold increase in ALP activity compared with the control group. This finding was confirmed again by quantitative analysis of ALP activity and by the results obtained via quantitative real-time polymerase chain reaction (qRT-PCR) assessments of ALP expression (Figure S2 C). Furthermore, higher concentrations of metformin (400 mg/mL and 1000 mg/mL) were evaluated; however, these concentrations did not yield a greater expression of ALP than that of the 200 mg/mL group (Figure S2 A, and B). A similar pattern was also observed in the ARS results (Figure S2 D). In summary, applying aMF containing metformin significantly enhanced the osteogenic potential of the tri-culture cells *in vitro*, and we have determined that a concentration of 200 mg/mL metformin was optimal for the bioink.

### 3.5 3D bio-printed aMF in CPC scaffold accelerated bone formation *in vivo*

**Fig. 5:**
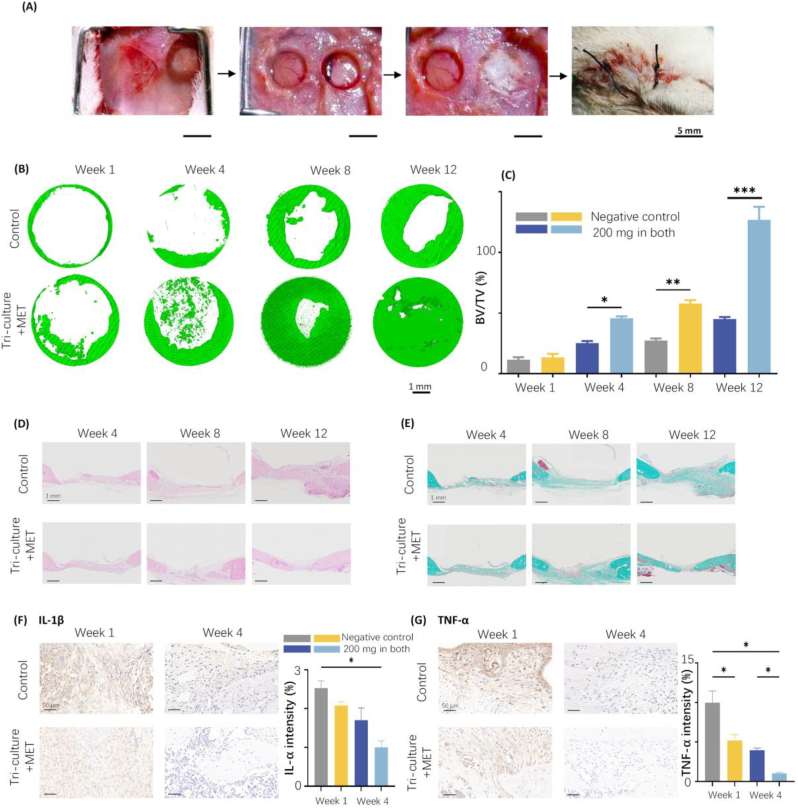
Osteogenic analysis of different 3D bio-printed aMF-CPC scaffold *in vivo*. (A) Schematic diagram of the rat bilateral cranial bone defect and the surgery process with control and scaffolds encapsulating tri-culture cells and metformin implanted. (Scale bar = 1 cm) (B) Micro-CT reconstruction of the bone defect area. (Scale bar = 1 cm) (C) Quantitative bone volume/tissue volume (BV/TV) analysis of the neo-bone within defect areas at 1, 4, 8, and 12 weeks. (n = 4) (D) HE staining of the neo-bone. (Scale bar = 1 mm) (E) Masson's trichrome staining of the neo-bone. (Scale bar = 1 mm) (F, G) Immunohistochemical staining of IL-1β and TNF-α (Scale bar = 50 μm) and corresponding semiquantitative statistics at weeks 1 and 4 post surgery. (n = 4).

The aMF-CPC scaffold's microstructural characteristics can function as a site for ossification in bone regeneration and significantly influence its biological functionality [36]. Rat cranial bone defect models were established to evaluate the neo-bone formation potential of the aMF-CPC scaffold, with the results compared to those of the negative control groups (Fig. 5A).

Animals were euthanized at 1, 4, 8, and 12 weeks postsurgery, and micro-CT scanning was conducted to assess the formation of neo-bone. The bone volume to total volume (BV/TV) ratio indicated that neo-bone formation in the aMF-CPC scaffold is significantly elevated, exhibiting a 2.5-fold increase compared to the negative control group (Fig. 5B–and C). The histological characteristics of the newly formed bone tissue were assessed using Hematoxylin and Eosin (HE) staining and Masson's trichrome staining techniques (Fig. 5D–and E). Notably, the groups of scaffolds encapsulating tri-culture cells and two-stage metformin exhibited well-regenerated neo-bone, in contrast to the control groups, demonstrating either low bone mass or immature bone regeneration. Consistent with micro-CT findings, a significant quantity of neo-bone formation was observed in the tri-culture and metformin groups. This observation verified the effective induction of osteogenesis promoted by seed cells and bioactive factors.

In addition, to assess the biocompatibility of the scaffold post-implantation, the expression levels of pro-inflammatory factors were analyzed. As demonstrated in Fig. 5F–and G, immunohistochemical staining for IL-1β and TNF-α underwent semiquantitative analysis using ImageJ software. The findings revealed that the CPC-aMF scaffold with tri-culture system and 200 mg/mL metformin group significantly reduced the expression of pro-inflammatory cytokines, thereby mitigating the inflammatory response.

These groups were tested: CPC-aMF scaffold without tri-culture cells or metformin as a negative control, CPC-aMF scaffold with tri-culture cells, and 200 mg/mL metformin in both CPC and aMF (controlled two-stage release of metformin). All values were presented as the mean ± SD, ∗*P* < 0.05, ∗∗*P* < 0.01, and ∗∗∗*P* < 0.001 analyzed by one-way ANOVA (n = 4).

### 3.6 Vascularization *in vivo*

Increasing evidence indicates that the capacity of scaffolds to promote angiogenesis is a significant factor in bone healing [[37], [38], [39]]. Developing tube-like structures by endothelial cells, such as hUVECs, is a crucial process in forming functional blood vessels. Various strategies have been employed to fabricate vessel-like structures, primarily by enhancing cell-cell and cell-extracellular matrix (ECM) interactions to reconstruct vascular morphology [40] effectively. Among these strategies, the tri-culture system represents a highly competitive approach. When interacting with PCs and hPDLSCs, hUVECs demonstrated a markedly enhanced ability to induce angiogenesis compared to the blank control group (CPC with 0 hPDLSCs, 0 MET blank).

**Fig. 6:**
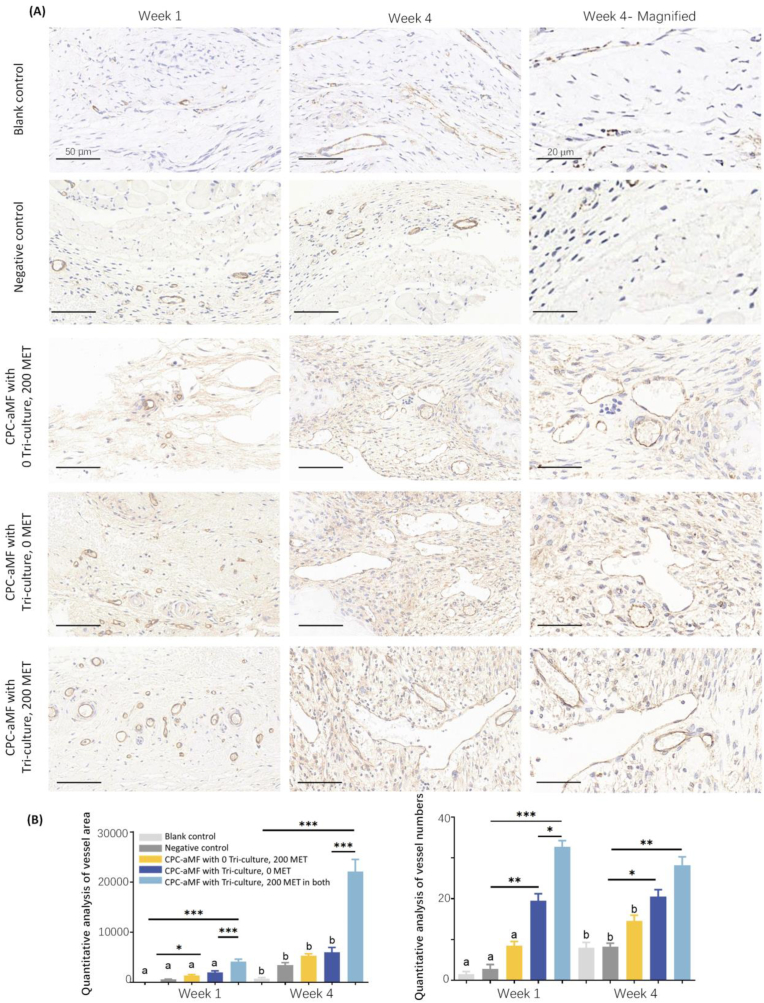
Histological analysis of vascularization in the 3D bio-printed aMF in CPC scaffold *in vivo*. (A) Immunohistochemical staining of CD31 at weeks 1 and 4, with magnified views of key regions of week 4. (Scale bar = 50 μm and 20 μm) (B) Corresponding semiquantitative statistics of vessel area and number at weeks 1 and 4 post surgery. (n = 4).

As illustrated in Fig. 6A–and B, the immunohistochemical staining for CD31 and α-SMA (Figure S3 A, and B) was subjected to semiquantitative analysis utilizing ImageJ. The results indicated that the formation of new vasculature in the aMF-CPC with tri-culture system and 200 mg/mL metformin group was observed to have more significant vessel area and vessel numbers than the other four groups at weeks 1 and 4. Compared to the blank control group, our novel 3D bio-printed aMF in the CPC scaffold group exhibited 20-fold higher vascular ingrowth. Meanwhile, it was 10 times greater than the negative control group. The tri-culture system could enhance the angiogenic capabilities of mono-cultured hUVECs. This phenomenon can be attributed to the interactions involving bioactive factors secreted by all three cell types.

These groups were tested: ①CPC scaffold without tri-culture cells encapsulated, and with 0 MET as blank control, ②CPC-aMF scaffold without tri-culture cells encapsulated, and with 0 MET as a negative control, ③CPC-aMF scaffold without tri-culture cells encapsulated, but with 200 mg/mL MET, ④CPC-aMF scaffold with tri-culture cells encapsulated, but with 0 MET, ⑤CPC-aMF scaffold with tri-culture cells encapsulated, and with 200 MET in both (200 mg/mL metformin in both CPC and aMF). All values were presented as the mean ± SD, ∗*P* < 0.05, ∗∗*P* < 0.01, and ∗∗∗*P* < 0.001 analyzed by one-way ANOVA (n = 4). Values with dissimilar letters are significantly different from each other (*P* < 0.05).

### 3.7 Innervation *in vivo*

The skeleton is a highly innervated structure characterized by the interaction between nerve fibers and various osteogenic lineage cells [41]. Recent studies have indicated that sympathetic innervation-mediated osteocytes play a crucial role in bone regeneration [42]. Interestingly, our results indicated that the presence of capillary-like tissue ingrowth was associated with an increased observation of nerve tissue within the bone defect region in the tri-culture group (CPC-aMF scaffold with tri-culture cells encapsulated and 100 MET in both).

**Fig. 7:**
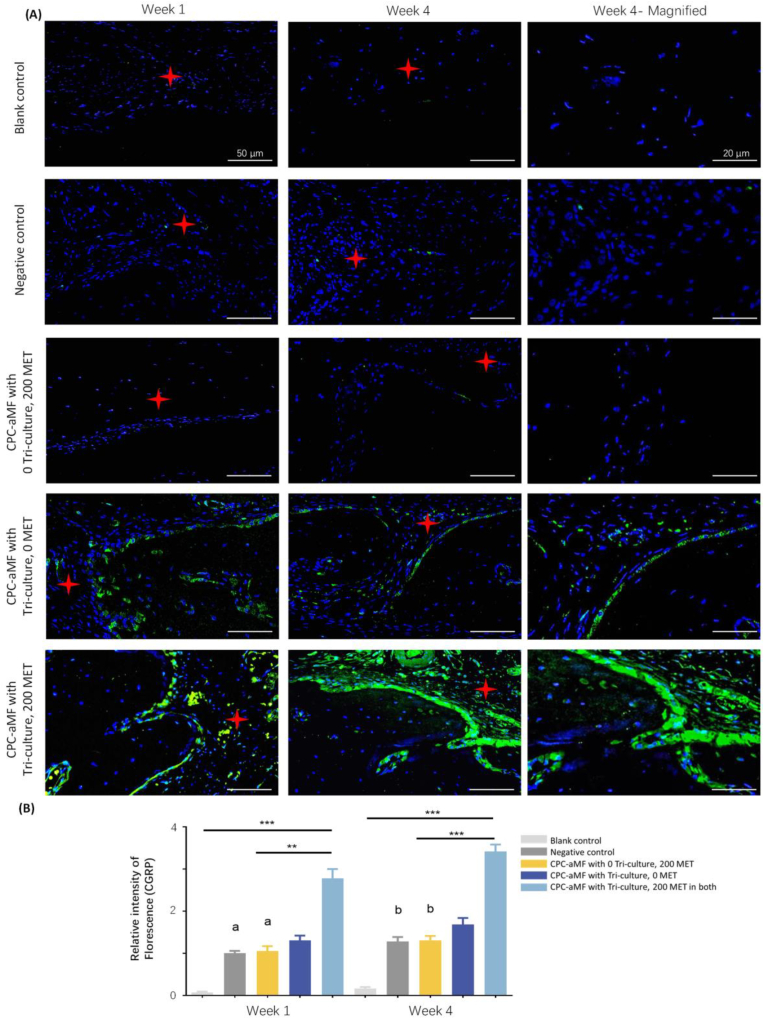
Histological analysis of innervation in the 3D bio-printed aMF in CPC scaffold *in vivo*. (A) Immunohistochemistry staining of CGRP at weeks 1 and 4, with magnified views of key regions of week 4. (Scale bar = 50 μm and 20 μm). (★ indicated the medial edge of the bone defect area) (B) Corresponding semiquantitative statistics of immunofluorescence intensity at weeks 1 and 4 postsurgery. (n = 4).

The scaffold demonstrated a significantly enhanced capacity for facilitating nerve in-growth compared to the negative control group during the initial phase of the study. As shown in Fig. 7A–and B, the immunofluorescence staining for Calcitonin Gene-Related Peptide (CGRP) and nestin-1 (Figure S4 A, and B) underwent semiquantitative analysis using ImageJ software. The findings demonstrated that the development of new nerve structures in the aMF-CPC with a tri-culture system and 200 mg/mL metformin group exhibited the highest fluorescence intensity compared to the other four groups during the early stages of weeks 1 and 4.

These groups were tested: ①CPC scaffold without tri-culture cells encapsulated, and with 0 MET as blank control, ②CPC-aMF scaffold without tri-culture cells encapsulated, and with 0 MET as a negative control, ③CPC-aMF scaffold without tri-culture cells encapsulated, but with 200 mg/mL MET, ④CPC-aMF scaffold with tri-culture cells encapsulated, but with 0 MET, ⑤CPC-aMF scaffold with tri-culture cells encapsulated, and with 200 MET in both (200 mg/mL metformin in both CPC and aMF). All values were presented as the mean ± SD, ∗*P* < 0.05, ∗∗*P* < 0.01, and ∗∗∗*P* < 0.001 analyzed by one-way ANOVA (n = 4). Values with dissimilar letters are significantly different from each other (*P* < 0.05).

**Fig. 8:**
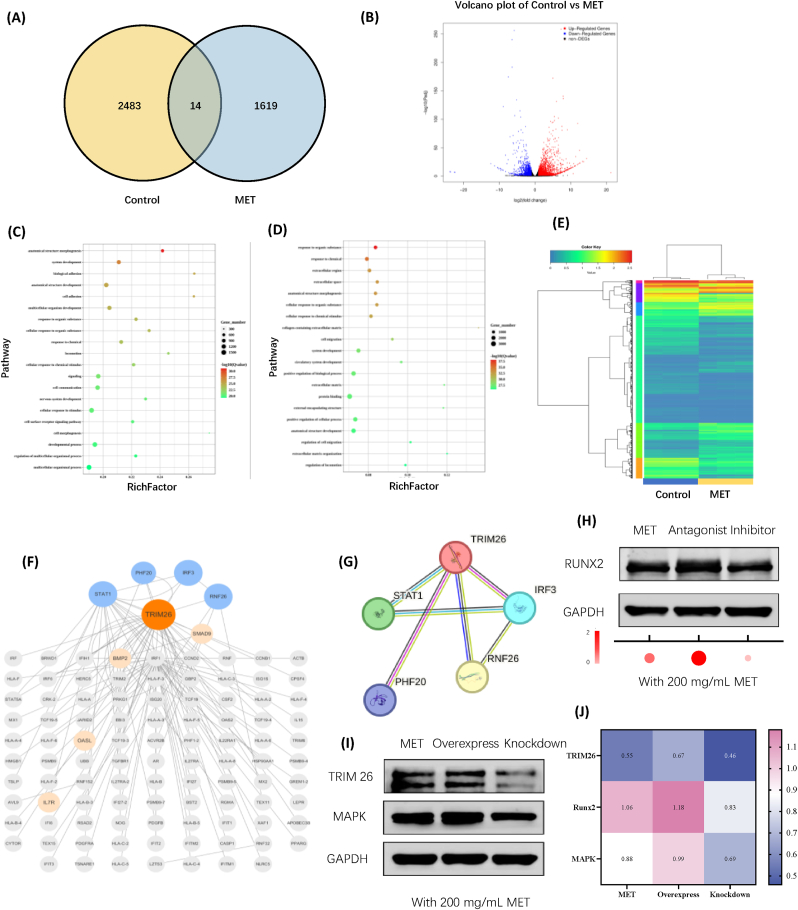
mRNA sequencing of tri-culture cells treated with metformin. (A) Venn diagram with numbers of genes up and down-regulated between the control and MET group. (B) A volcano plot illustrating the results of RNA sequencing. In this representation, genes that are upregulated are indicated in red, those that are downregulated in blue, and genes that remain unchanged in black. (C, D) GO term analysis for differentially expressed genes and proteins. (E) Heatmap analysis of differentially expressed genes. (F, G) Protein-protein interaction (PPI) networks are associated with key signalling pathways. (H–J) Changes and quantitative analysis of western blot assays evaluating the expression levels of the associated proteins.(For interpretation of the references to colour in this figure legend, the reader is referred to the Web version of this article.)

We analyzed the functional gene and protein profiles of the tri-culture cells to elucidate the molecular mechanisms by which metformin influences the tri-culture system. This analysis was conducted for both the negative control and 200 mg/mL metformin groups, utilizing RNA sequencing techniques. The analysis demonstrated significant differences in the expression profiles of genes and proteins. Specifically, metformin showed a significant upregulation of 2483 genes and a downregulation of 1619 genes when compared to those groups without metformin (*P* < 0.05 and log2FC > 1.0), resulting in a total of 4102 genes regulated (Fig. 8A). Additionally, a volcano plot was used to display the distribution (Fig. 8B).

To achieve a deeper comprehension of the functional implications associated with genes that exhibit differential expression, a gene ontology (GO) term enrichment analysis was performed. The GO term results indicated that metformin could enhance osteogenic differentiation in hPDLSCs-based tri-culture cells by promoting the activation of various enriched biological pathways [28]. These included, in particular, the "cell communication" aspect of the tri-culture system (Fig. 8C–and D). Furthermore, a heatmap was also conducted to visually illustrate the expression patterns of the differentially expressed genes (Fig. 8E).

The metformin group exhibited elevated expression levels of specific genes associated with bone formation processes compared to the control group. The influential genes identified in this study (the protein-protein interaction analysis) included Tripartite Motif Containing 26 (TRIM26), STAT1, IRF3, RNF26, and PHF20 (Fig. 8F–and G). E3 ligase-TRIM26 might play a role in suppressing both the self-renewal ability and the *in vivo* tumorigenic potential of glioblastoma stem cells [43]. Moreover, E3 ligase-TRIM might also be a crucial regulator in maintaining homeostasis in dopaminergic neurons [44]. These might indicate that the tri-culture system may facilitate nerve in growth during the bone regeneration process through the involvement of the TRIM26 gene.

Nonetheless, changes in the targeted gene (as identified through RNA sequencing), along with the expression of potentially related proteins, necessitate further experimental validation to establish a causal relationship. Consequently, to validate the potential involvement of TRIM26 in the bone regeneration process, western blot analysis was employed. The metformin tri-culture system was treated with Forskolin, a cAMP/MAPK-TRIM26 antagonist [45], and Nutlin-3, a p53/MDM2-TRIM26 inhibitor [46,47]. Additionally, transfection with shRNA plasmids was conducted to facilitate both the overexpression and knockdown of TRIM26 within the tri-culture system (Figure S5, A and B). Following this, protein extraction and collection were performed. The results from the western blot analysis indicated that levels of TRIM26, Runx2, and MAPK were significantly elevated in the antagonist and overexpression groups, while significantly reduced in the inhibitor and knockdown groups (Fig. 8H–and J). The quantification of the resulting bands was analyzed using ImageJ software (Figure S6 A, and B). These results indicate a possible correlation between TRIM26 and the cAMP-MAPK signalling pathways in the regulation of bone tissue repair processes.

These groups were tested: Control (without tri-culture cells and with 0 mg/mL metformin), MET (with tri-culture cells and with 200 mg/mL metformin), Agonist (with tri-culture cells, 200 mg/mL metformin and with 100 μM Forskolin), Inhibitor (with tri-culture cells, 200 mg/mL metformin and with 20 μM Nutlin-3), Overexpress (with tri-culture cells, 200 mg/mL metformin and with 4 μg overexpress shRNA plasmid), Knockdown (with tri-culture cells, 200 mg/mL metformin and with 4 μg knockdown shRNA plasmid). All values were presented as the mean ± SD, ∗*P* < 0.05, ∗∗*P* < 0.01, and ∗∗∗*P* < 0.001 analyzed by one-way ANOVA (n = 4).

### 3.8 Future clinical translation potential of the scaffolds

The lack of innervation in numerous clinical situations of bone defects is frequently associated with a reduction in both structural and functional recovery [48]. Notably, the repair of alveolar bone defects and jaw reconstruction are the most significant issues in the field of dental bone repair. The implantation of our innovative CPC-aMF scaffolds into clinical practice offers the potential to promote innervation in bone defects, thereby stimulating osteogenesis and improving the overall quality of life for patients.

Neuro-bone tissue engineering represents a novel strategy for the treatment of critical-sized bone defects [49]; however, its application in clinical practice remains constrained. The primary challenge lies in the complex nature of the surgical procedures involved in tissue repair. Additionally, the economic implications of the product construction must be considered, as culturing three distinct primary cell types entails more than tripling efforts compared to a single cell type. While the biocompatibility of the scaffolds has been rigorously evaluated through both *in vitro* and *in vivo* studies, the potential side effects associated with exogenous scaffolds should not be disregarded.

Nevertheless, despite these obstacles, the successful regeneration of tissue-engineered bone through our scaffolds remains feasible, when appropriate seed cells (tri-culture system), factors (metformin), and the CPC-aMF scaffold are carefully selected.

## 4 Conclusions

We developed an hPDLSCs-based tri-culture system for the first time. This tri-cultured cellular framework has the capacity to promote bone, vasculature and nerve regeneration simultaneously. Based on the tri-culture system, we have successfully constructed an innovative 3D-printed microfibers encapsulating stem cells in scaffold with tri-culture and two-stage metformin release. Within the tri-culture system, the complex dynamics of interaction among the three cells-hPDLSCs, hUVECs and PCs-facilitated a conducive microenvironment for the regeneration of triple tissues. Notably, the E3 ligase TRIM may be significantly involved in these mechanisms. This 3D bio-printed aMF in CPC scaffold promoted new bone regeneration, with 2.5-fold more significant new bone amount *in vivo* than the negative control (CPC-aMF with 0 tri-culture cells, 0 MET control), and 4.5-fold more incredible new bone amount than blank control (CPC with 0 tri-culture cells, 0 MET blank). Furthermore, the observed increases in vascular and nerve ingrowth were 3-fold and 3.5-fold, respectively, compared to the negative control group. These findings suggest that our innovative scaffold holds significant promise for future applications in the repair of critical-sized bone defects.

## CRediT authorship contribution statement

**Minjia Zhu:** Writing – original draft, Visualization, Methodology, Formal analysis, Data curation. **Xinyi Li:** Investigation. **Le Xiao:** Investigation. **Kan Yu:** Investigation. **Jingyi Li:** Investigation. **Zixiang Dai:** Investigation. **Qinrou Zhang:** Investigation. **Jialiang Dai:** Investigation. **Zihan Jia:** Investigation. **Yuxing Bai:** Writing – review & editing, Supervision, Resources, Funding acquisition. **Ke Zhang:** Writing – review & editing, Supervision, Resources, Funding acquisition, Conceptualization.

## Ethics approval and consent to participate

Our study was approved by Animal Ethical and Welfare Committee of Beijing Stomatological Hospital affiliated to Capital Medical University (SYXK:2023-0052), and the Ethics Committee of Beijing Stomatological Hospital affiliated to Capital Medical University（0000-0001-7982-5758).

## Declaration of competing interest

The authors declare that they have no known competing financial interests or personal relationships that could have appeared to influence the work reported in this paper.
