# Supplementary material for: 3D-printed microfibers encapsulating stem cells in scaffold with tri-culture and two-stage metformin release for bone/vasculature/nerve regeneration in rats
Source: Bioact Mater. 2025 May 21;51:399–413. doi: 10.1016/j.bioactmat.2025.05.011 (PMC12148644; doi:10.1016/j.bioactmat.2025.05.011)
Supplement: Multimedia component 2 [file mmc2.docx]

Supporting Information

3D-printed microfibers encapsulating stem cells in scaffold with tri-culture and two-stage metformin release for bone/vasculature/nerve regeneration in rats

**This PDF file includes:**

Figs. S1 to S6

Tables S1 to S2

Movies S1

**Other Supplementary Materials for this manuscript include the following:**

Movies S1

**Fig. S1.**


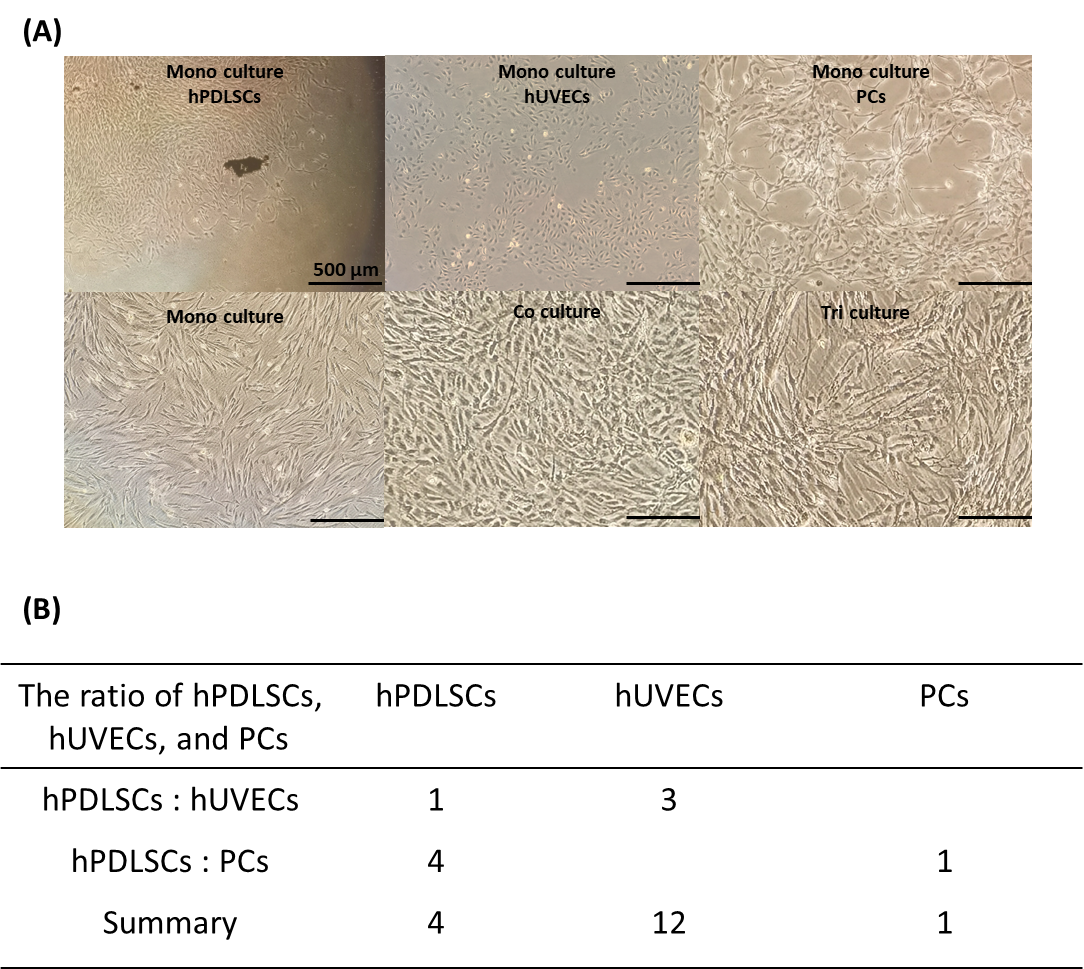


**Fig. S1. The ratio design of the hPDLSCs-hUVECs-PCs triculture system.** (A) Microscopic images of mono-cultured, co-culture and tri-culture hPDLSCs, hUVECs and PCs. Scale bar = 500 μm. (B) Table of the ratio design. Scale bar = 500 μm.

**Movie S1. (separate file)**

**
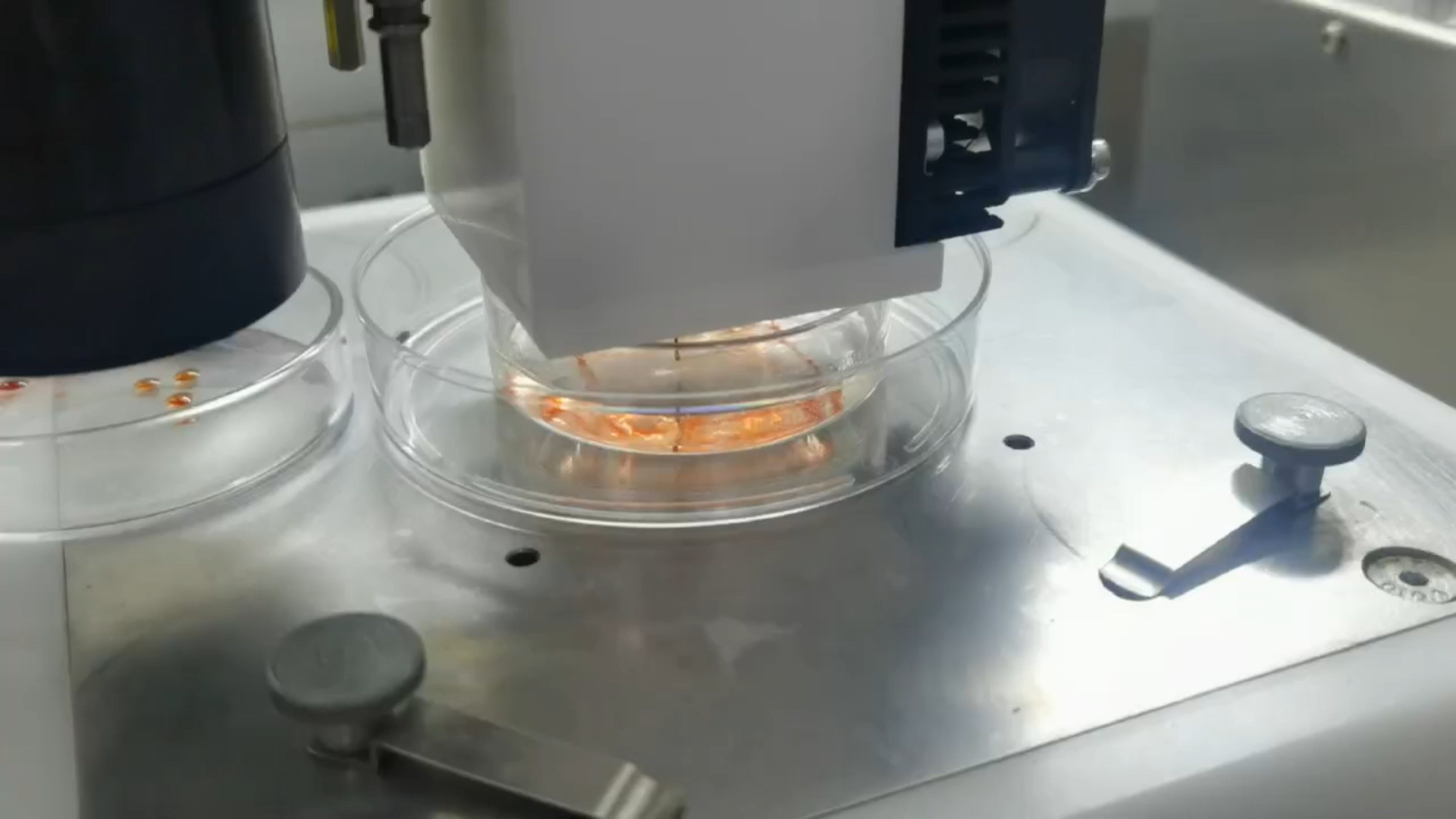
**

**Movie S1. 3D bio-printing microfiber MF.** (**A**) 3D bio-printing microfiber following the FRESH bath strategy.

**Fig. S2.**


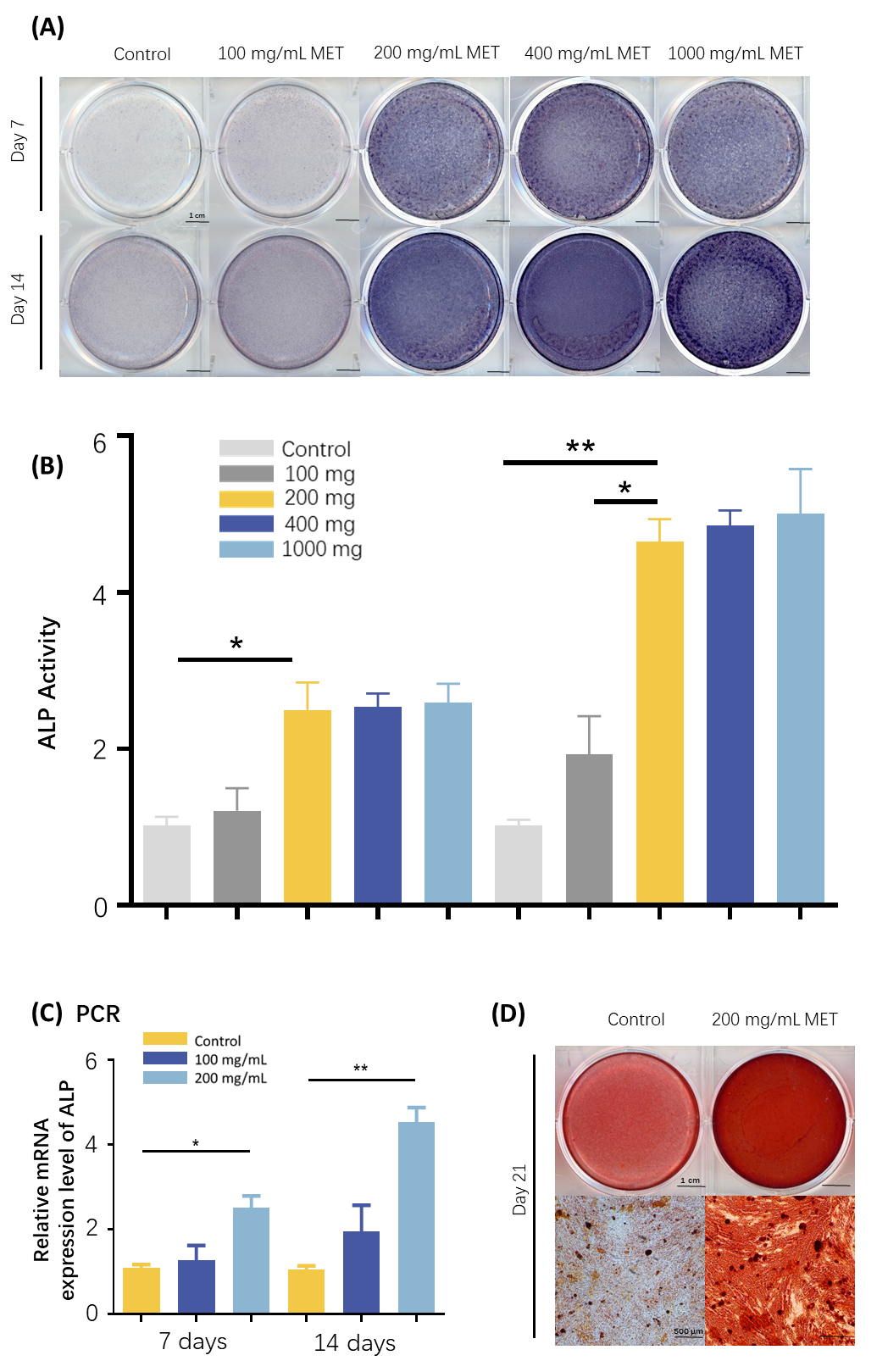


**Fig. S2. ALP staining of triculture system with different concentration of metformin.** (**A**) ALP staining of tri-cultured cells with 0 mg, 100 mg, 200 mg, 400 mg and 1000 mg metformin after 7 and 14 days of culture. (B) Semi-quantification of ALP activity of triculture system with 0 mg, 100 mg, 200 mg, 400 mg and 1000 mg metformin after 7 and 14 days of culture. (C) Quantitative ALP expression determined by qRT-PCR at Day 7 and 14. (D) ARS staining of tri-cultured cells with 0 mg, 200 mg/mL metformin after 21 days of culture. All values were presented as the mean ± SD, **P* < 0.05, ***P* < 0.01, and ****P* < 0.001 analyzed by one-way ANOVA (n = 4).

**Fig. S3.**


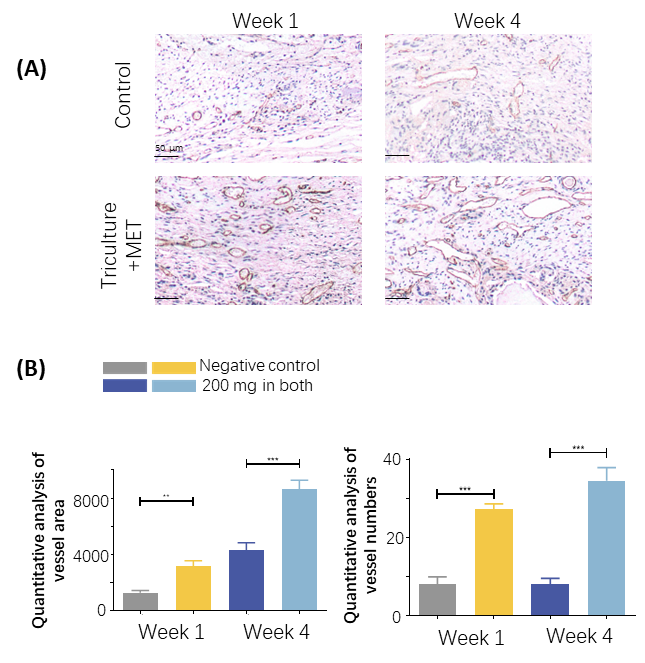


**Fig. S3. Histological analysis of vascularization in the 3D bio-printed aMF in CPC scaffold *in vivo*.** (A) Immunohistochemical staining of α-SMA at week 1 and 4. (Scale bar = 50 μm) (B) Corresponding semiquantitative statistics of vessel area and number at week 1 and 4 post surgery. (n = 4) All values were presented as the mean ± SD, **P* < 0.05, ***P* < 0.01, and ****P* < 0.001 analyzed by one-way ANOVA (n = 4).

**Fig. S4.**


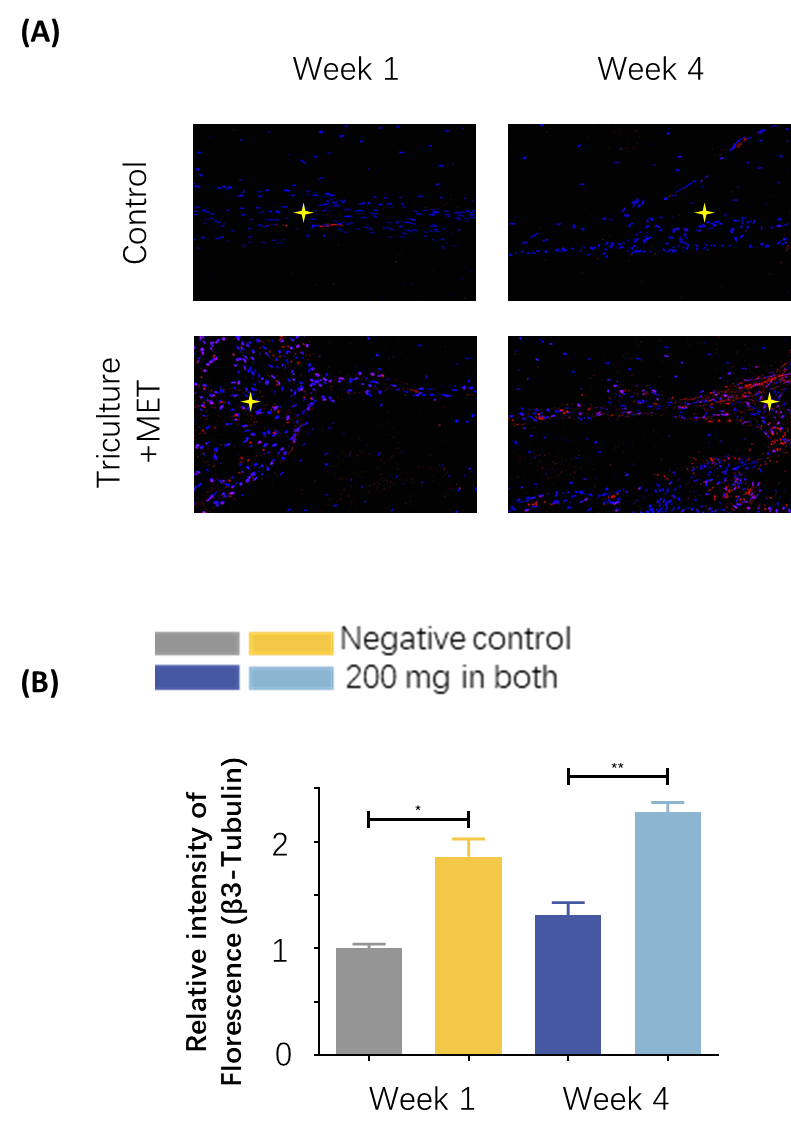


**Fig. S4. Histological analysis of innervation in the 3D bio-printed aMF in CPC scaffold *in vivo*.** (A) Immunohistochemistry staining of β3-Tubulin at week 1 and 4. (Scale bar = 50 μm). (★ indicated the medial edge of the bone defect area) (B) Corresponding semiquantitative statistics of immunofluorescence intensity at week 1 and 4 postsurgery. (n = 4). All values were presented as the mean ± SD, **P* < 0.05, ***P* < 0.01, and ****P* < 0.001 analyzed by one-way ANOVA (n = 4).

**Fig. S5.**


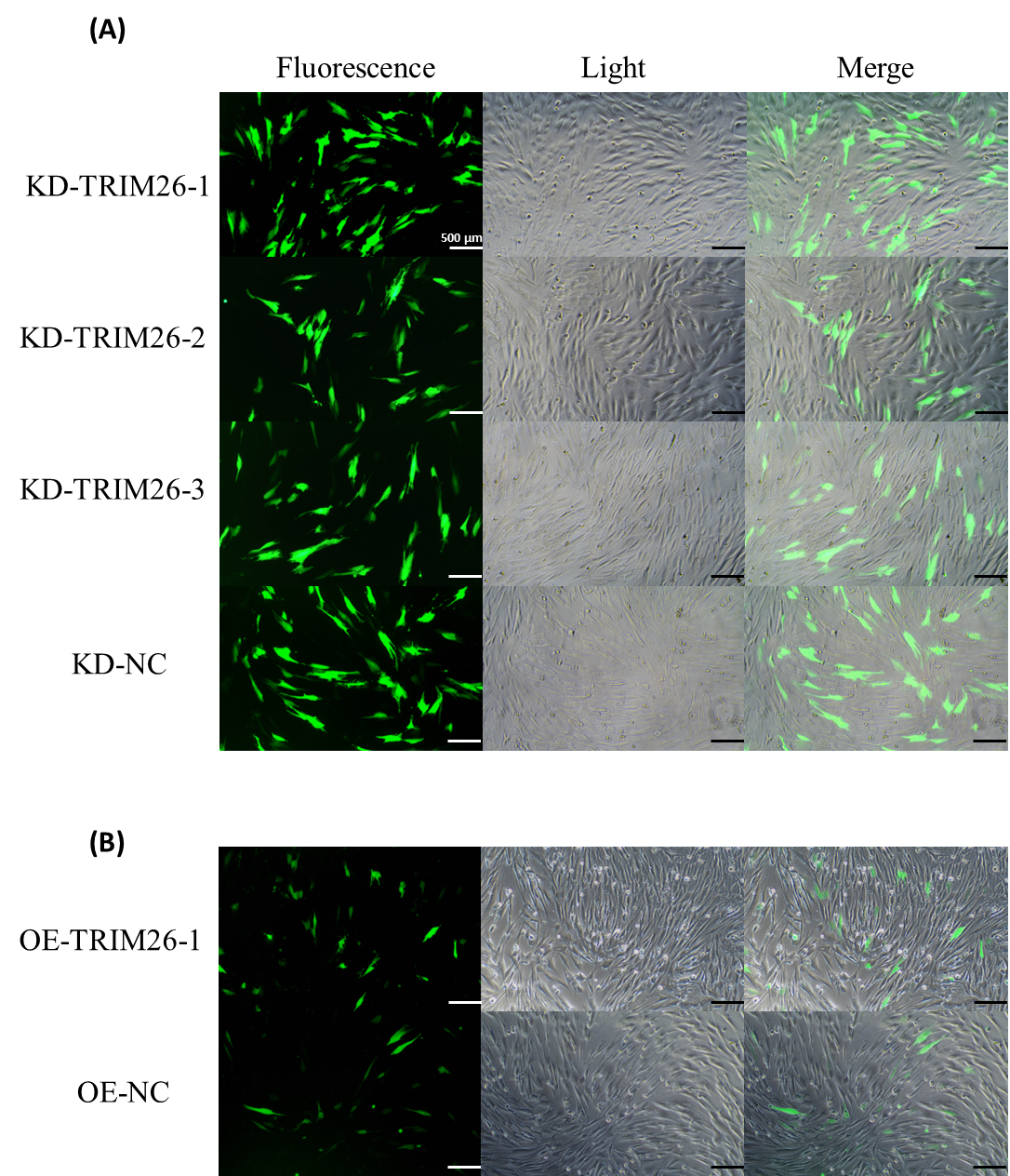


**Fig. S5. Fluorescence microscopic and light microscopic images of TRIM26 gene knockdown and overexpress by plasmid transfection.** (**A**) TRIM26 gene knockdown (B) TRIM26 gene overexpress. (Scale bar = 200 μm)

**Fig. S6.**


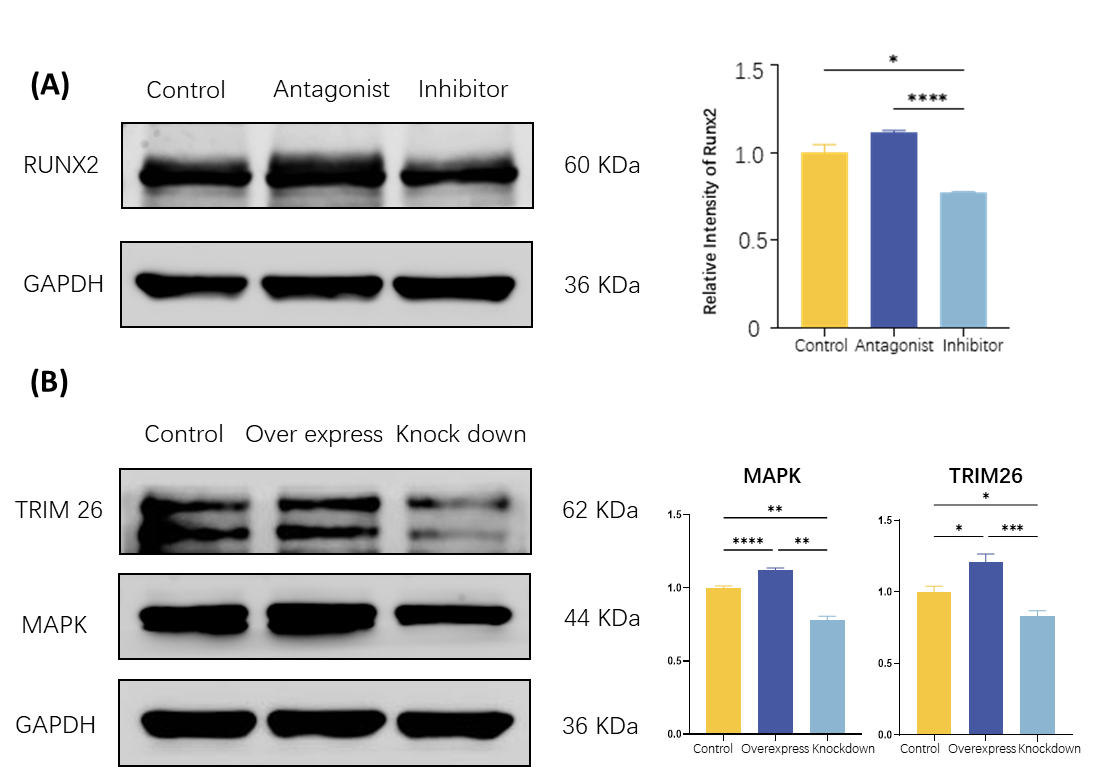


**Fig. S6. Western blotting images and semi-quantification of Runx2, TRIM26, and MAPK.** All values were presented as the mean ± SD, *P < 0.05, **P < 0.01, and ***P < 0.001 analyzed by one-way ANOVA (n = 4).

**Table S1.**

**Table S1. Preparation of the bioink for 3D bio-printing MF.**

**
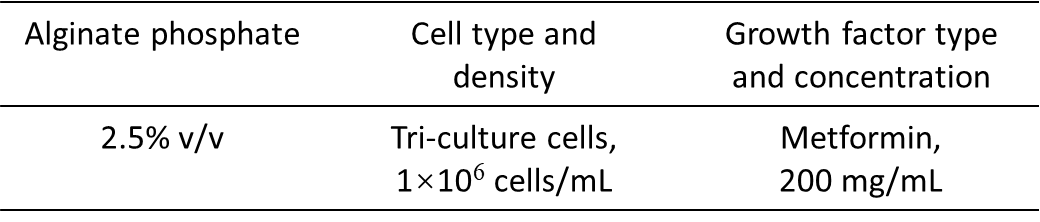
**

**Table S2.**


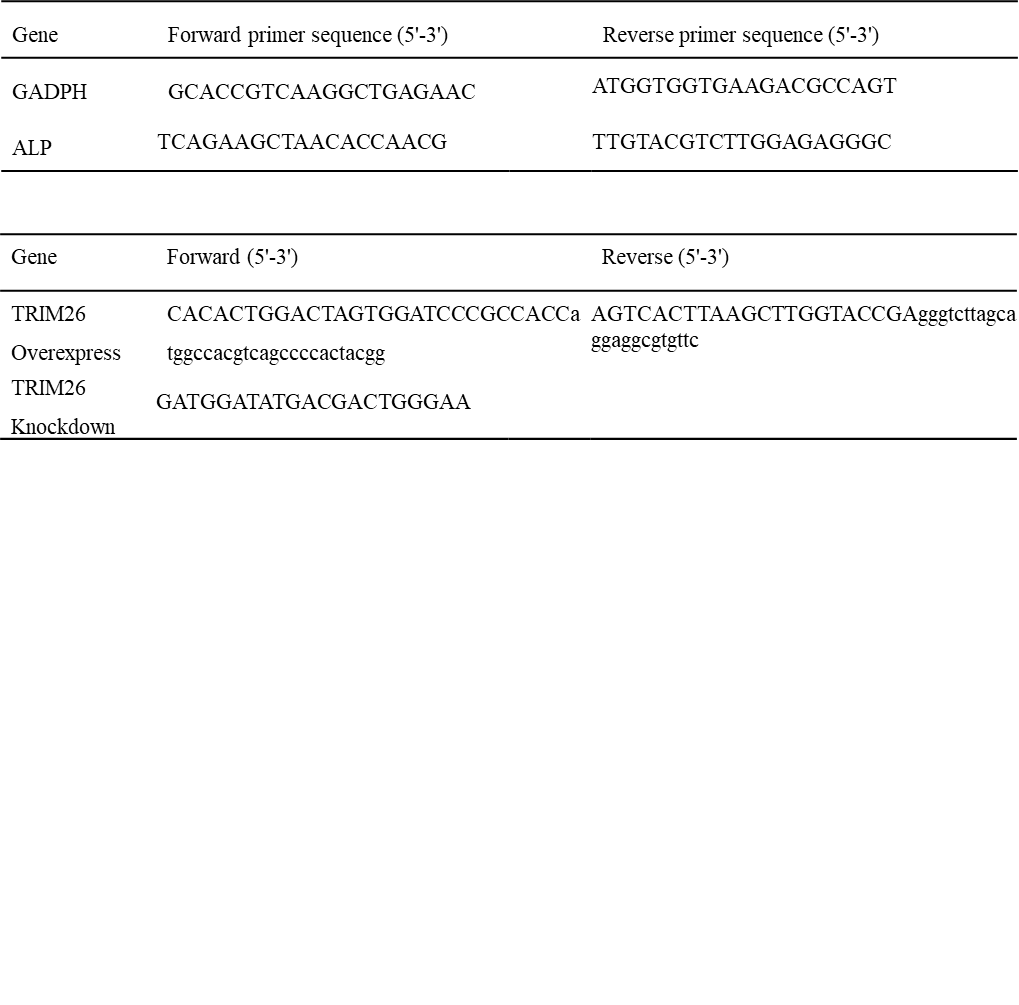


**Table. S2. Sequences of primers for RT-PCR experiments and TRIM26 plasmid gene.** (A) The sense and antisense PCR primers. (B) TRIM26 plasmid gene for knockdown and overexpress.
